# Mechanical Behavior and Constitutive Modeling of the Mg-Zn-Y Alloy in an Electrically Assisted Tensile Test

**DOI:** 10.3390/ma15207203

**Published:** 2022-10-15

**Authors:** Zhichao Xu, Wenju Yang, Jianfeng Fan, Tao Wu, Zeng Gao

**Affiliations:** 1 School of Materials Science and Engineering, Henan Polytechnic University, Jiaozuo 454003, China; 2 Key Laboratory of Interface Science and Engineering in Advanced Materials, Ministry of Education, School of Materials Science and Engineering, Taiyuan University of Technology, Taiyuan 030024, China

**Keywords:** Mg-Zn-Y alloy, flow stress, electroplastic effect, arrhenius model

## Abstract

The Mg-Zn-Y alloy containing the LPSO phase has excellent mechanical properties and functional application prospects. In an effort to clarify the electrically assisted deformation behavior of the Mg-Zn-Y alloy, electrically assisted tensile tests of Mg_98.5_Zn_0.5_Y_1_ alloy sheets were carried out at different temperatures, current densities, duty ratios, and frequencies. The experimental results showed that, after the pulse current was applied (26.58 A·mm^−2^), the peak stress of the sample deformed at 200 °C decreased by 8 MPa. The peak stress of the material decreased with the increase in current density. It is noticeable that the changes in duty ratios and frequencies have a small effect on the peak stress and strain. When the current was applied, more recrystallized grains appeared in the alloy and the basal texture was weakened. According to the experimental results, the Arrhenius model was derived based on the Zener–Hollomon parameter. Owing to the appearance of the stacking fault structure (LPSO), the activation energy Q of the Mg_98.5_Zn_0.5_Y_1_ alloy was 389.41 KJ/mol, which is higher than conventional Mg alloys. Moreover, the constitutive equation of the electro plastic effect coupled with temperature and pulse current parameters was established by introducing electrically assisted characteristics. By comparing the experimental and predicted values, the established model can effectively predict the variation trend of flow stress under electrically assisted deformation. Moreover, the constitutive model was incorporated into the UHARD subroutine of ABAQUS software to study the deformation behavior of the Mg_98.5_Zn_0.5_Y_1_ alloy.

## 1. Introduction

Magnesium alloys exhibit great potential in aerospace, military, automobile, and biomedical applications thanks to their low density, high specific strength, and good recyclability [1]. In the last decade, Mg-Zn-Y alloys have received much attention owing to their unique microstructures of long-period stacking order (LPSO) phases and significant improved mechanical properties [2]. It is well-known that there are three types of ternary phases in the Mg-Zn-Y alloy system: I phase (Mg_3_Zn_6_Y), W phase (Mg_3_Zn_3_Y_2_), and LPSO phase [3]. These three phases play an important role in improving the mechanical properties of the alloy and the application of functionalization [4,5]. In particular, the LPSO phase can effectively hinder the movement of dislocations. However, the close hexagonal structure and poor deformation ability restrict the large-scale application of the alloy in the industrial field. Therefore, hot deformation is an important processing method for magnesium alloys [6,7,8,9]. Although the elevated-temperature forming methods are often applied for Mg-Zn-Y alloys [10], it has a few noticeable drawbacks, such as lubrication between the workpiece and die, multiple annealing process, and the wastage of die. Various studies have shown that the existence of continuous current during metal plastic deformation can significantly reduce the flow stress of metal [11,12]. Although there is no conclusive explanation on the mechanism of the additional effect of current, the existence of related phenomena has been generally recognized. The phenomenon of electroplasticity is very attractive to researchers and industries in the metal forming field [13,14] and it has a large application potential.

Salandro et al. [15] studied the electrically assisted uniaxial tension of aluminum alloy. Under the condition of selective pulse current, the elongation of the alloy increased by 190%. Ross et al. [16] investigated the effect of current on aluminum alloy, titanium alloy, and stainless steel; the results showed that current significantly affects the flow stress and elongation. The electroplastic effect (EPE), which is the phenomenon that electric current reduces the flow stress and increases the ductility of materials, could be employed to assist the deformation of alloys [17]. Kim et al. [18] proposed an empirical expression describing the electroplastic deformation behavior of AZ31 magnesium alloy and Al-Mg-Si alloy, and the model can effectively describe the tensile deformation behavior under pulse current. Roh et al. [19] studied the electro-plastic effect of aluminum and put forward an empirical expression to describe the tensile behavior of aluminum alloy under pulse current. The empirical expression included three electro plastic coefficients: material constant, electric energy density, and electric pulse period. Li et al. [20] established a semi-phenomenological model for predicting the current-carrying dynamic deformation behaviors of aluminum alloy and the model exhibited preferable prediction accuracy. Compared with the traditional processing technology, the electrically assisted processing only needs to add a pulse power supply and contact electrode to the equipment and insulate some parts of the equipment. The requirements for hardware facilities are not high and the cost is relatively low. Although studies have been conducted on the constitutive modeling, as mentioned above, quantitative descriptions of the electroplastic effect on Mg-Zn-Y alloys are still limited. The Mg-Zn-Y alloy is a promising high-strength wrought Mg alloy. If electroplastic processing technology can be introduced into the processing of the Mg-Zn-Y alloy, it will help to improve the theoretical system of the electroplastic effect in magnesium alloy processing and promote green development in the field of hard deformation metal processing.

In the present work, the tensile properties of Mg-Zn-Y alloy under different temperatures, current densities, duty ratios, and frequencies are systematically studied. The microstructure evolution and dislocation annihilation during electro-assisted uniaxial tension are observed. The influence of the electroplastic effect on the deformation behavior and microstructure of materials is analyzed. The effect of electric pulse parameters on the stress–strain behavior of the Mg-Zn-Y alloy is discussed by comparing the experimental results of high-temperature and electrically assisted uniaxial tension. In addition, the constitutive equation describing the electroplastic deformation behavior is established based on the Arrhenius model by adding electrical parameters.

## 2. Experimental Materials and Procedures

### 2.1. Experimental Materials

**Figure 1:**
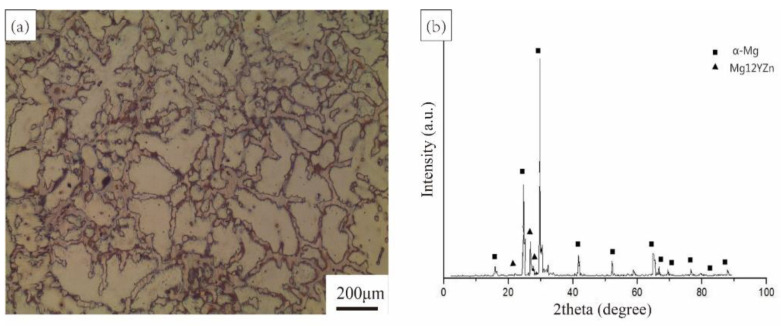
(**a**) OM image and (**b**) XRD pattern of the Mg_98.5_Zn_0.5_Y_1_ alloy.

Samples with the nominal composition Mg_98.5_Zn_0.5_Y_1_ (at. %) were prepared from a high-purity Mg, Zn, and Mg-25Y (wt.%) master alloy by high-frequency induction melting in a graphite crucible at approximately 1023 K under an argon atmosphere. The composition of the alloy ingot was measured by an X-ray fluorescence spectrometer. The chemical composition of the alloy is shown in Table 1. The microstructure was examined using optical microscopy (OM, Carl Zeiss Axio Observer, Jena, Germany). X-ray diffractometry (XRD) was carried out in a θ-2θ diffractometer Philips 1810 (Almelo, Dutch) using Cu Kα radiation, voltage of 35 kV, and current of 50 mA. Figure 1 shows the microstructure of the Mg_98.5_Zn_0.5_Y_1_ alloy. It can be seen that the average grain size is 146 μm. The primary phase of the Mg_98.5_Zn_0.5_Y_1_ alloy is Mg_12_YZn (LPSO).

### 2.2. Experimental Procedures

**Figure 2:**
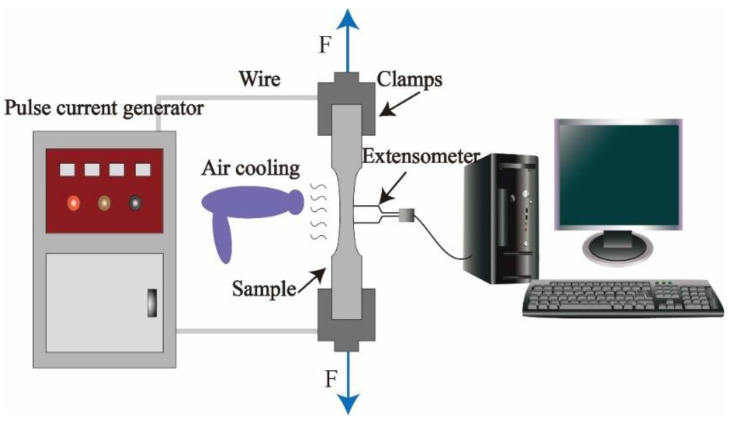
Schematic diagram of the electrically assisted tensile test.

To better analyze the non-Joule thermal effect, the isothermal uniaxial tensile test was carried out at the same time. These samples were uniaxially stretched under elevated temperature and electrically assisted conditions. According to relevant references [21], the sets of experimental parameters are listed in Table 2. The isothermal and electrically assisted uniaxial tensile tests were carried out on a SANS testing machine. The electric current was created using a TSGZ-2.0KVA (Junke, Linyi, China) pulse power supply, which can adjust the current, duty ratio, and frequency in real time. Besides, the power supply can measure the numerical current of these parameters in real time through an oscilloscope. In the electrically assisted tensile test, two ends of the sample were clamped on the copper electrode of the pulse power supply. The temperature increase of samples during stretching was monitored by thermal imager. To overcome the inaccuracy of infrared temperature measurement, the surface of the sample was evenly sprayed with black paint. When the temperature reaches the specified temperature, the tensile test of the Mg-Zn-Y alloy will begin. The temperature was controlled by the blower within the specified temperature range, and the error of temperature shall not exceed 5 °C. To avoid damage of the pulse current to the universal material testing machine, an insulating gasket was added inside the tensile fixture to ensure sufficient insulation between the sample and the testing machine, as shown in Figure 2.

In the process of electrically assisted stretching, the sample was continuously elongated and the cross section of the sample decreased with the increase in deformation. To maintain the constant current density in the sample, the output current of the power supply is adjusted in real time according to the deformation degree of the sample. The true current density j can be calculated as follows:

(1) $$j=I/\left(W_{0}t\right)$$

where *j* is current density (A· mm^−2^); *I* is current; *W*_0_ is the gauge width of the sample; and t is the thickness of the sample (mm).

## 3. Results and Discussion

### 3.1. Flow Stress Behavior

**Figure 3:**
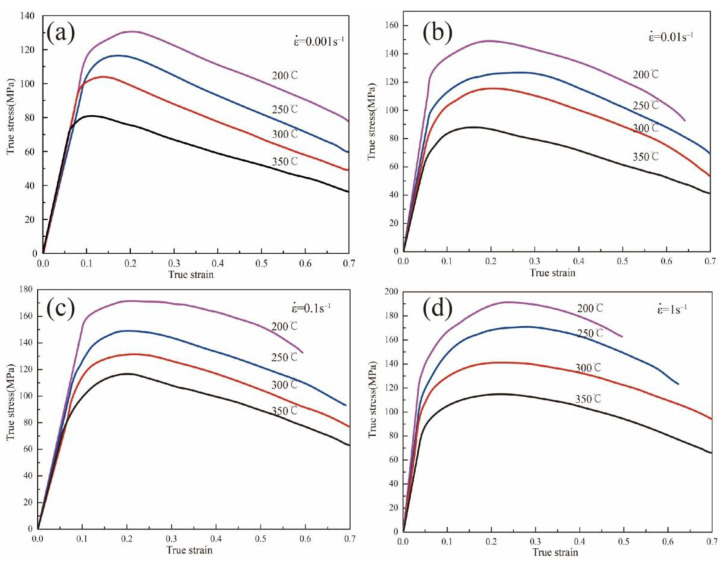
True stress–strain curves of the Mg_98.5_Zn_0.5_Y_1_ alloy at various temperatures and strain rates. (**a**) $\dot{\varepsilon }=0.001$ s^−1^; (**b**) $\dot{\varepsilon }=0.01$ s^−1^; (**c**) $\dot{\varepsilon }=0.1$ s^−1^; (**d**) $\dot{\varepsilon }=1$ s^−1^.

The flow stress curves at various temperatures and strain rates obtained from isothermal tensile tests are shown in Figure 3. It can be seen from the figure that the flow stress curve under each deformation condition presents roughly the same change trend. It can be divided into three different stages: hardening stage, transition stage, and softening stage [22]. In the initial stage of deformation, the flow stress increases linearly with the increase in strain. This phenomenon is explained by work hardening caused by the generation and accumulation of dislocation. In the transition stage, with the increase in the deformation degree, dislocations will accumulate continuously. When the deformation of the alloy reaches the critical deformation and a certain temperature, dynamic recrystallization (DRX) will occur [23]. It is worth noting that magnesium alloys are prone to DRX during isothermal deformation because of the low stacking fault energy [24]. With the increase in dynamic recovery and recrystallization degree, the work hardening and dynamic softening reach the dynamic equilibrium stage at a smaller strain degree, that is, the higher the deformation temperature, the smaller strain required for the dynamic equilibrium of the flow stress. These results agree with those reported by Wu et al. [25]. 

By comparing the stress–strain curves under different deformation conditions, it can be found that the deformation temperature and strain rate are sensitive factors affecting the flow behavior of the Mg_98.5_Zn_0.5_Y_1_ alloy. Under the same strain rate, the peak stress in the curve increases gradually with the decrease in temperature. For example, when the strain rate is 0.001 s^−1^, the peak stress of the sample deformed at 200 °C is 128 MPa, while the peak stress of the sample deformed at 250 °C is 117 MPa. With regard to the same deformation temperature, the peak stress in the curve increases gradually with the increase in the strain rate. It can be drawn from Figure 3a,b that, when the temperature is 200 °C, the peak stress of the sample deformed at 0.001 s^−1^ is 128 MPa, while the peak stress of sample deformed at 0.01 s^−1^ is 151 MPa. Taking the above observations into account, we conclude that the flow stress increases with a higher strain rate, while the flow stress decreases with an increasing temperature.

**Figure 4:**
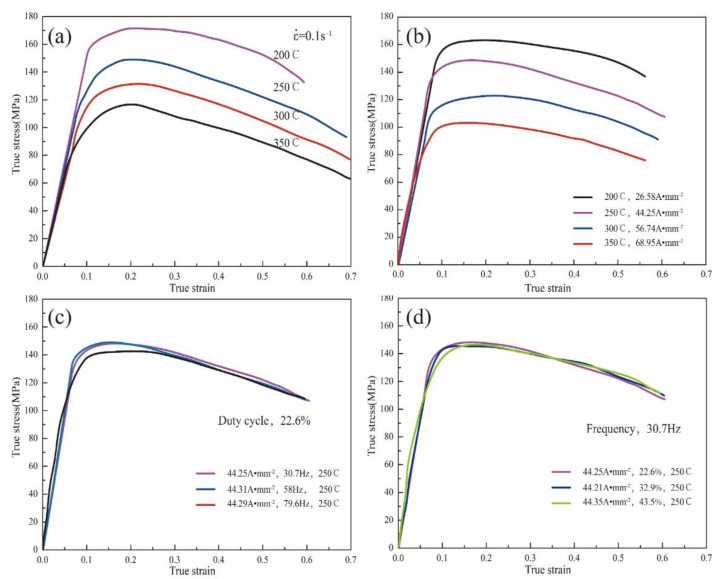
True stress–strain curves of the Mg_98.5_Zn_0.5_Y_1_ alloy subjected to electrically-assisted tests. (**a**) $\dot{\varepsilon }=0.1$ s^−1^; (**b**) pulse current density; (**c**) pulse frequency; (**d**) duty ratio.

The true stress–strain curves of the Mg_98.5_Zn_0.5_Y_1_ alloy under different temperatures, current densities, duty ratios, and frequencies are shown in Figure 4. The true stress–strain curve of the alloy at a strain rate of 0.1 s^−1^ with different deformation temperatures (200 °C, 250 °C, 300 °C, and 350 °C) is shown in Figure 4a. It is noted that, with the increase in the deformation temperature, the peak stress of the material decreases gradually. Figure 4b shows the true stress–strain curve under different current densities with duty ratio (22.6%) and frequency (30.7 Hz). With the increase in current density, the peak stress of the material decreases gradually. By contrasting the stress–strain curves with and without current, we can obtain that the reduction in the peak stress of the sample is higher than the sample without pulse current at the same temperature. For example, as shown in Figure 4a,b, when the current is not applied, the peak stress of the sample deformed at 200 °C is 169 MPa. After the pulse current (26.58 A·mm^−2^) is applied, the peak stress of the sample deformed at 200 °C is 161.2 MPa, and the value of the peak stress decreased by 8 MPa. When the pulse current was maintained at 68.95 A·mm^−2^, in contrast with the samples deformed at 350 °C, the value of the peak stress decreased by 17 MPa. Our findings lead us to conclude that the effect of current density on the flow stress is evident. It is worth noting that the elongation of the material also decreases. The reason is that, when the current is passed, the current density will increase in a local range, aggravating the inhomogeneous temperature of the material. The inhomogeneity of temperature will promote the further expansion of necking and reduce the elongation of the material.

Figure 4c shows the true stress–strain curve of the alloy under different current frequencies with pulse current density (≈44 A·mm^−2^) and duty ratio (22.6%). At the frequencies of 30.7 Hz and 58 Hz, the peak stresses of the material are 148 MPa and 149.2 MPa, respectively. The peak stresses of the two are almost the same. Figure 4d shows the true stress–strain curve of the alloy under different duty ratio with pulse current density (≈44 A·mm^−2^) and current frequencies (30.6 Hz). The minor change in the stress–strain curve leads us to conclude that the duty ratio has a small effect on the flow stress and strain of the Mg_98.5_Zn_0.5_Y_1_ alloy.

### 3.2. Microstructure of Samples after the Tensile Test

**Figure 5:**
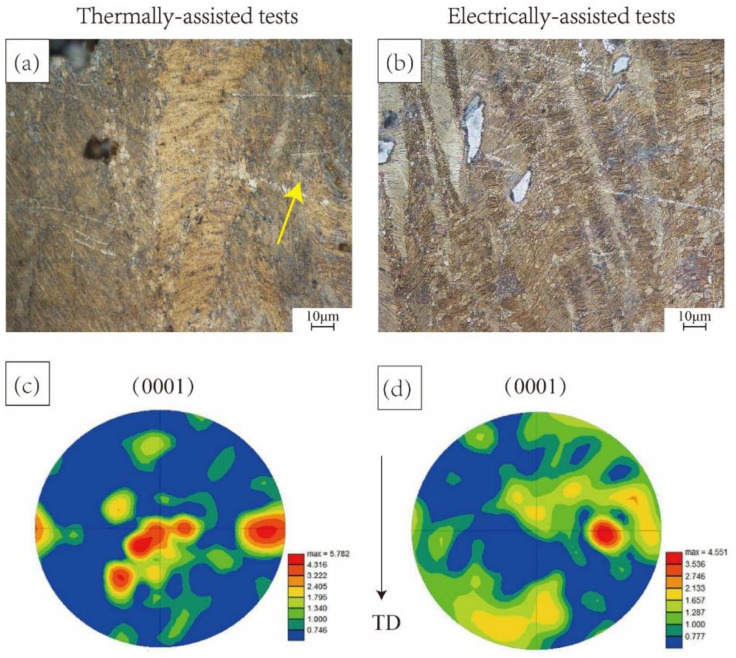
Microstructure and pole figures of the alloy (**a**,**c**) thermally assisted tests (200 °C) and (**b**,**d**) electrically assisted tests (26.58 A∙mm^−2^).

Figure 5 shows the microstructure of the alloy. As shown in Figure 5a, when the tensile test is conducted at 200 °C without current, there are more twins and various grain types can be observed in the microstructure, as indicated by yellow arrows. When pulse current (26.58 A∙mm^−2^) is applied, as shown in Figure 5b, it is obvious that there is a larger range of recrystallized grains than in the isothermal structure. This indicates that the non-thermal effect enhances the nucleation of recrystallized grains and accelerates the movement of dislocation and slip. This will help magnesium alloys open new slip systems, facilitate cross slip, and greatly reduce flow stress. By comparing the base plane pole figure, as exhibited in Figure 5c,d, it can be found that the pulse current during the electrically assisted tensile test can reduce the base plane orientation of the sample. The reason was attributed to the thermal and non-thermal coupling effect, which increases the driving force of recrystallization and nucleation rate. This will result in complete recrystallization at a low temperature, which consequently led to the weakened base plane orientation. The change in microstructure is coincident with the result of the mechanical properties.

**Figure 6:**
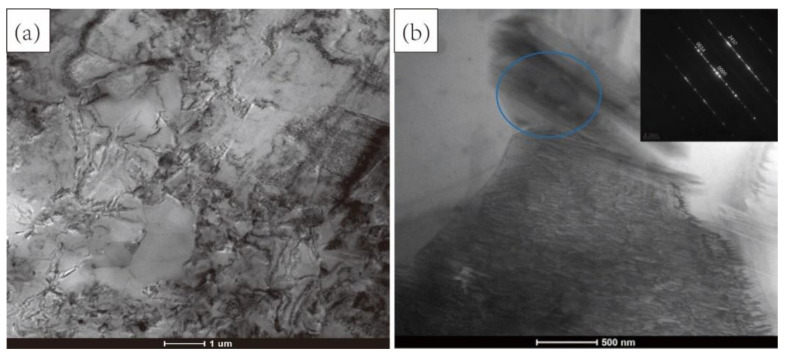
(**a**) TEM images of electrically assisted sample (26.58 A∙mm^−2^); (**b**) the corresponding selected area electron diffraction (SAED) of the area marked circle.

In order to distinguish the deformed grains and to further reveal the microstructural features of the Mg_98.5_Zn_0.5_Y_1_ alloy, the TEM images are shown in Figure 6. It can be seen that there are many subgrains in the alloy. This indicates that the pulse current accelerates the formation of subgrains. The addition of current does not change the phase structure in the alloy, which is still LPSO (marked by blue circle). These LPSO phases are dispersed in the matrix. Our previous research reported that these long-period ordered structures can block the movement of dislocations and improve the mechanical properties of the alloy [4]. In this experiment, when the current is applied, the electron wind generated by the collision of high-speed electrons with the atomic nucleus is beneficial to the mobility of dislocations and increases the speed of dislocation climb [13]. This theory can also be used to explain why the tensile strength of magnesium alloy containing LPSO phase decreases during the process of electrically assisted tension.

### 3.3. Establishment of Constitutive Equation

The above experimental results describe the effects of electrical parameters such as current density, duty ratio, and pulse frequency on the mechanical properties of the Mg_98.5_Zn_0.5_Y_1_ alloy at different temperatures. In the present study, based on the high temperature constitutive equation, we construct a functional expression including electrical parameters:

(2) $$\dot{\varepsilon }=f\left(\sigma \right)\cdot h\left(j,f,d\right)$$

where $\dot{\varepsilon }$ is the strain rate; f(σ) is the constitutive model of Mg_98.5_Zn_0.5_Y_1_ alloy; and h (j,f,d) is electro plastic effect function. The constitutive model is obtained by high-temperature tensile test and the electroplastic effect function, which can be drawn from the electrically assisted tensile test. 

In the present study, firstly, the thermal constitutive equations will be constructed. It is widely approved that thermal deformation is a process controlled by thermal activation and the flow stress of materials is very sensitive to the deformation temperature and strain rate [26]. Usually, the constitutive equation of flow stress in the process of plastic deformation can be established based on the Arrhenius equation, which possesses high simplicity and accuracy in describing the relationship among deformation temperature, strain rate, and flow stress. Sellars and Mctegard [27] proposed a modified Arrhenius relationship including deformation activation energy Q and temperature T:

(3) $$\dot{\varepsilon }=A{\left[\sinh\left(\alpha \sigma \right)\right]}^{n}\exp\left(-\frac{Q}{RT}\right)$$

(4) $$\dot{\varepsilon }=A_{1}\sigma^{n_{1}}\exp\left(-\frac{Q}{RT}\right)$$

(5) $$\dot{\varepsilon }=A_{2}\left[\exp\left(\beta \sigma \right)\right]\exp\left(-\frac{Q}{RT}\right)$$

Equation (3) describes the high-temperature deformation of metal flow stress under all stress conditions; Equation (4) can be applied to low stress (*ασ* < 0.8); Equation (5) can be applied to high stress conditions (*ασ* > 1.2); *Q* is the activation energy of deformation (KJ/mol^−1^), which is an important physical parameter acting as an indicator of deformation difficulty in the plasticity deformation theory [28]; $\dot{\varepsilon }$ is the strain rate; R is the universal gas constant (8.314 J/K); σ is the peak stress (MPa); n is the stress index (1/m); T is the absolute temperature (K); and *A*, *A*_1_, *A*_2_, *N*_1_, *α*, and *β* are the material constants. The stress multiplier a can be approximated as $\alpha =\beta /n_{1}$. Based on Equations (3)–(5), some constants can be derived as below:

(6) $$n_{1}={\left[\frac{\partial \ln\dot{\varepsilon }}{\partial \ln\sigma_{p}}\right]}_{T},\beta ={\left[\frac{\partial \ln\dot{\varepsilon }}{\partial \sigma_{p}}\right]}_{T}$$

(7) $$n={\left[\frac{\partial \ln\dot{\varepsilon }}{\partial \ln\left\{\sinh\left(\alpha \sigma_{p}\right)\right\}}\right]}_{T},m={\left[\frac{\partial \ln\left\{\sinh\left(\alpha \sigma_{p}\right)\right\}}{\partial \left(1/T\right)}\right]}_{\dot{\varepsilon }}$$

**Figure 7:**
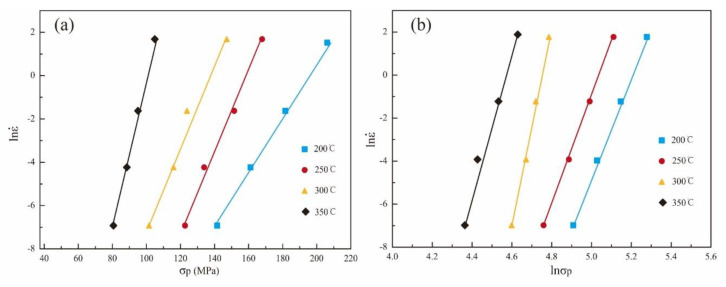
Relationship between flow stress and strain rate of the alloy: (**a**) ln$\dot{\varepsilon }$ − *σ* and (**b**) ln$\dot{\varepsilon }$ − ln*σ*.

By fitting the $\ln\dot{\varepsilon }-\ln\sigma$ and $\ln\dot{\varepsilon }-\sigma$, the values of *β* and n_1_ are obtained, respectively. In this study, the relationship between the flow stress and the strain rate (Figure 7) leads us to conclude that the value of *α* is 0.0112.

**Figure 8:**
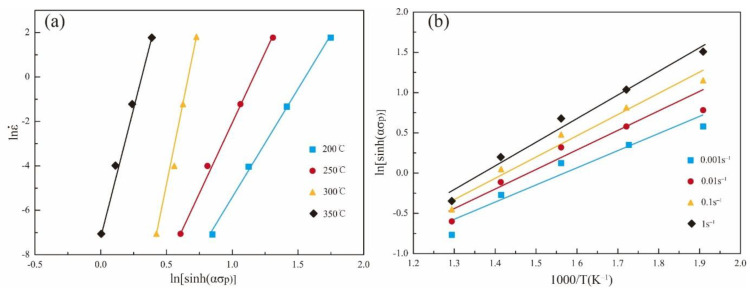
Relationship between flow stress and strain rate or temperature: (**a**) ln$\dot{\varepsilon }$ − ln[sinh(*ασp*)] and (**b**) ln[sinh(*ασp*)] − 1000/T.

To establish the relationship among the flow stress, strain rate, and deformation temperature, the activation energy of the deformation (Q) of the alloy needs to be calculated. Taking the average of the slopes obtained by fitting the data in Figure 8, the values of n and m are 16.15 and 2.94, respectively. According to the above relationship, the activation energy can be expressed as follows:

(8) $$Q=R{\left[\frac{\partial \ln\dot{\varepsilon }}{\partial \ln\left\{\sinh\left(\alpha \sigma_{p}\right)\right\}}\right]}_{T}{\left[\frac{\partial \ln\left\{\sinh\left(\alpha \sigma_{p}\right)\right\}}{\partial \left(1/T\right)}\right]}_{\dot{\varepsilon }}=Rnm$$

The thermal activation energy can be calculated, which is 389.41 KJ/mol, by taking the values of R, n, and m into Formula (8). What is surprising is that the activation energy of this alloy is significantly higher than other conventional Mg alloys. For example, the activation energy of the Mg-Ca alloy is 100–200 KJ/mol [29] and the activation energy of the Mg-Al-Zn alloy is 125–132 KJ/mol [30]. It is worth noting that similar values of activation energy (on the order of 200–300 KJ/mol) have been previously reported for many cast Mg-RE-Zn alloys [31,32]. It has been pointed out that the higher activation energy of the Mg-Zn-Y alloy is due to the LPSO phase, which act as a barrier to effectively hinder dislocation motion [33].

In order to facilitate the calculation, the deformation temperature and strain rate can be combined into one parameter, which is the Zener–Hollomon parameter (Z). The Z parameter is an important index of microstructure evolution during thermal deformation, and it is an important parameter to measure the influence of temperature and strain rate on thermal deformation behavior [34]. The hyperbolic sine function incorporated with the Z parameter is shown in Equation (9).

(9) $$Z=\dot{\varepsilon }\exp\left(\frac{Q}{RT}\right)=A{\left[\sinh\left(\alpha \sigma \right)\right]}^{n}$$

In order to simplify the calculation, natural logarithms are taken on both sides of Equation (9), as shown in Equation (10).

(10) $$\ln Z=\ln A+n\ln\left[\sinh\left(\alpha \sigma_{p}\right)\right]$$

**Figure 9:**
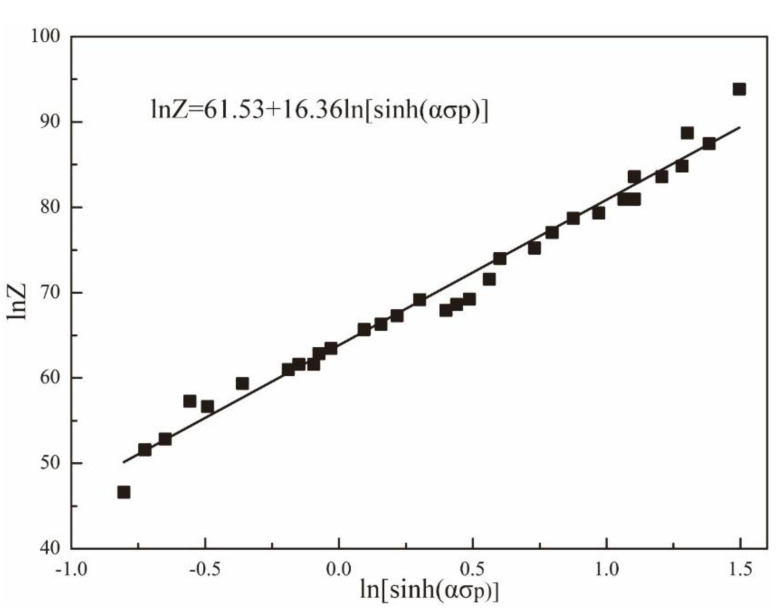
Relationship between ln [sinh (*ασp*)] and lnZ.

Equation (10) shows that there is a linear relationship between lnZ and ln [sinh (*ασ_p_*)], as shown in Figure 9. It can be clearly seen that the value of *n* is the slope of the regression curve and the value of lnA is the intercept of the regression curve on the vertical axis. According to the linear regression calculation, the value of *n* is 16.36, which is close to the results (*n* = 16.15) of Figure 6. This indicates that the relationship between flow stress, deformation temperature, and strain rate can be effectively described by the Arrhenius relationship of this alloy. Moreover, based on the linear regression calculation, the value of A is 7.0152 × 10^26^.

Finally, by substituting the calculated value of the parameters A, *n*, *α*, and *Q* into Equation (3), the thermal constitutive equation of Mg_98.5_Zn_0.5_Y_1_ alloy can be obtained as follows:

(11) $$\dot{\varepsilon }=7.0152\times 10^{26}{\left[\sin h\left(0.0112\sigma \right)\right]}^{16.15}\exp\left(\frac{-389410}{RT}\right)$$

Then, the electroplastic effect function will be established. According to the results in Figure 4, we can conclude that the current density has a significant influence on the mechanical properties of the material, while the duty ratio (d) and current frequency (f) do not. Therefore, the electroplastic effect function is only determined by the pulse current density, and it can be written as follows:

(12) h(j,f,d)=h(j)

Molotskii [35] found that the improvement in metal plasticity is brought about by the facilitation of dislocation depinning caused by the current-induced magnetic field. Moreover, Molotskii put forward a model considering the electroplastic effect, caused by the action of the magnetic field, induced by the electric current. The model can be written as a function of the current:

(13) $$\sigma \left(j\right)=\sigma \left(0\right)\cdot \left(1-\frac{j^{2}}{j^{2}+j_{0}^{2}}\right)$$

where σ(j) is the effective stress when pulse current is applied; σ(0) is the effective stress without pulse current; and *j*_0_ is the material constant. It is pointed out that *j*_0_ is related to the temperature of the material. Therefore, the electroplastic effect function is defined as follows:

(14) $$h\left(j\right)=1-\frac{j^{2}}{j^{2}+j_{0}^{2}\left(T\right)}$$

By fitting the data of the electrically assisted tensile test, the values of $j_{0}^{2}\left(T\right)$ are obtained, as shown in Table 3.

**Figure 10:**
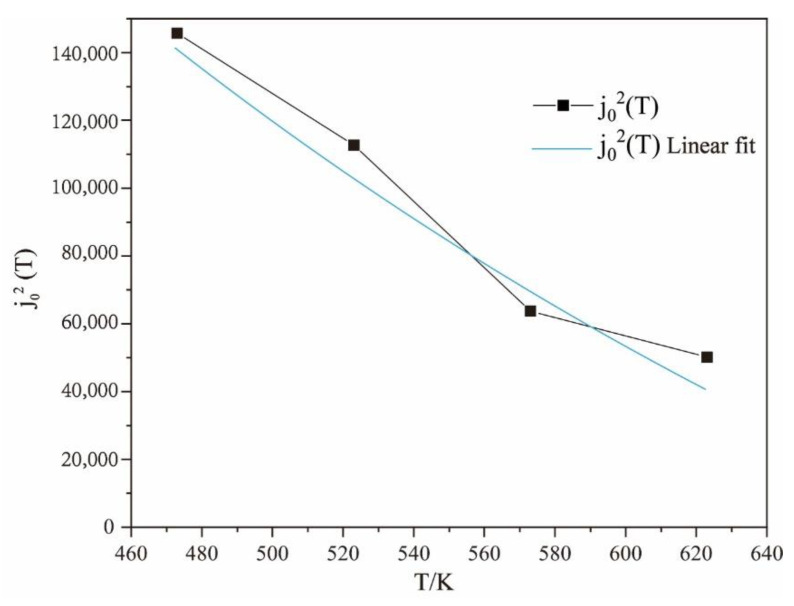
$j_{0}^{2}\left(T\right)$ of the Mg_98.5_Zn_0.5_Y_1_ alloy at different temperatures.

From Table 3, it can be deduced that the value of $j_{0}^{2}\left(T\right)$ eventually tends to 0 with the increase in temperature. The $j_{0}^{2}\left(T\right)$ equation of the test alloy can be fitted by an exponent function, as shown in Figure 10. Therefore, the $j_{0}^{2}\left(T\right)$ can be calculated as follows:

(15) $$j_{0}^{2}\left(T\right)=3.15\times 10^{6}\exp\left(-8.74\times 10^{-3}T\right)$$

In the end, combined with the thermal constitutive equation and the electroplastic effect function, the constitutive equation of the Mg_98.5_Zn_0.5_Y_1_ alloy coupling with flow stress, temperature, and pulse current parameters can be obtained as follows:

(16) $$\dot{\varepsilon }=7.0152\times 10^{26}{\left[\sin h\left(0.0112\sigma \right)\right]}^{16.15}\exp\left(\frac{-389410}{RT}\right)\cdot \left[1-\frac{j^{2}}{j^{2}+3.15\times 10^{6}\exp\left(-8.74\times 10^{-3}T\right)}\right]$$

**Figure 11:**
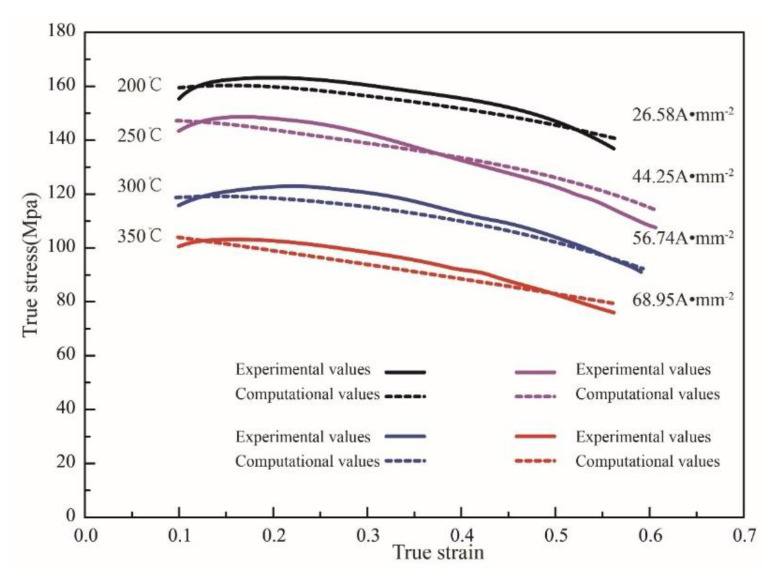
Comparison between calculated values and experimental values of flow stress.

To verify the accuracy of the constitutive equation established in this paper, the corresponding test conditions are brought into Equation (16) and the calculated results are compared with the experimental data. As shown in Figure 11, the maximum relative error between the calculated results and the experimental values is 8.4% and the average relative error is 5.12%. This indicates that the constitutive equation coupled with pulse current parameters established in this paper can accurately predict the flow stress of the Mg_98.5_Zn_0.5_Y_1_ alloy under the electrically assisted tensile test.

### 3.4. Finite Element Model and Simulation Results

**Figure 12:**
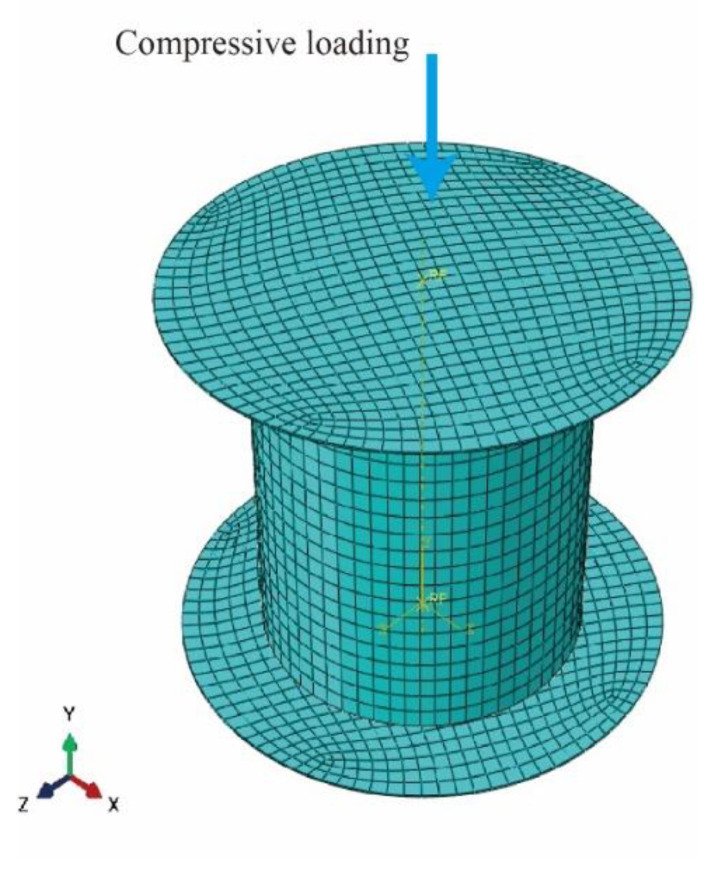
Grid model of the sample.

In the present study, the commercial ABAQUS simulation software was used to simulate and reproduce the hot compression process. Figure 12 shows the grid model of the sample. The upper and lower punches are modeled by discrete rigid bodies and reference points are set up respectively. The direction of extrusion is shown by the blue arrow in the figure. The simulation was carried out at 200 °C, under strain rates of 0.001 s^−1^ and 1 s^−1^.

It is worth noting that the Arrhenius constitutive model for strain compensation is not included in ABAQUS, so the UHARD user subroutine is used to implement this constitutive model in this simulation. In the UHARD user subroutine, there are four parts: SYIELD, HARD (1), HARD (2), and HARD (3), which need to be defined by the user [36]. Actually, SYIELD represents the yield strength, while HARD (1), HARD (2) and HARD (3) stand for the partial derivatives of yield strength corresponding to strain, strain rate and temperature, respectively. The expressions of these variables can be written as follows:

(17) $$\mathrm{SYIELD}\;=\sigma =\frac{1}{\alpha \left(\varepsilon \right)}{\sin h}^{-1}\left[{\left(\frac{Z\left(\varepsilon \right)}{A\left(\varepsilon \right)}\right)}^{\frac{1}{n\left(\varepsilon \right)}}\right]$$

(18) HARD(1)=0

(19) $$\begin{array}{cc} \mathrm{HARD}\left(2\right) & ={\left[\frac{\partial \sigma }{\partial \dot{\varepsilon }}\right]}_{T}\\ & =\frac{{\sin h}^{1-n\left(\varepsilon \right)}\left[\left(\alpha \left(\varepsilon \right)\sigma \right)\right]}{\alpha \left(\varepsilon \right)A\left(\varepsilon \right)n\left(\varepsilon \right)\sqrt{1+{\sin h}^{2}\left[\left(\alpha \left(\varepsilon \right)\sigma \right)\right]}}\exp\left[\frac{Q\left(\varepsilon \right)}{RT}\right] \end{array}$$

(20) $$\mathrm{HARD}\left(3\right)={\left[\frac{\partial \sigma }{\partial T}\right]}_{\dot{\varepsilon }}=\frac{-Q\left(\varepsilon \right)\sin h\left[\left(\alpha \left(\varepsilon \right)\sigma \right)\right]}{\alpha \left(\varepsilon \right)n\left(\varepsilon \right)RT^{2}\sqrt{1+{\sin h}^{2}\left[\left(\alpha \left(\varepsilon \right)\sigma \right)\right]}}$$

**Figure 13:**
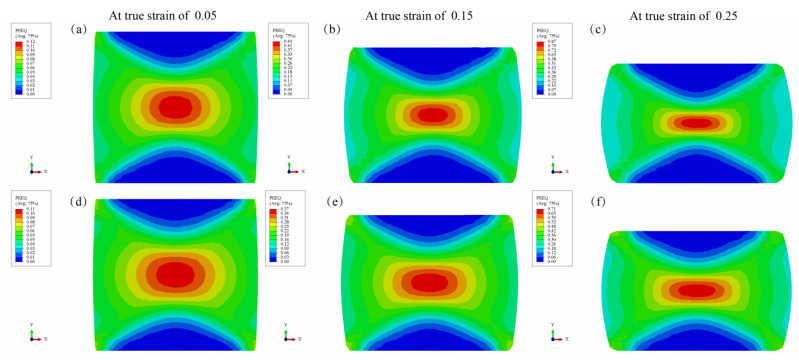
Equivalent plastic strain distribution of the Mg_98.5_Zn_0.5_Y_1_ alloy under different deformation conditions: (**a**–**c**) 200 °C and 0.001 s^−1^; (**d**–**f**) 200 °C and 1 s^−1^.

Figure 13 shows the equivalent plastic strain distribution of the Mg_98.5_Zn_0.5_Y_1_ alloy under different deformation conditions. It can be seen that the equivalent plastic strain at the center of the samples deformed at 200 °C and 0.001 s^−1^ is higher than the one deformed at 200 °C and 1 s^−1^. The equivalent plastic strain distribution of the whole specimen is not uniform. This is mainly due to the friction between the punch and the sample surface during compression, which is consistent with a previous investigation of the Mg-8.5Gd-4.5Y-0.8Zn-0.4Zr alloy [37].

**Figure 14:**
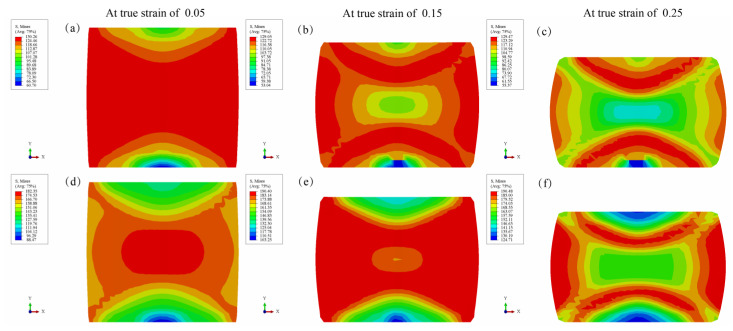
Stress distribution of the Mg_98.5_Zn_0.5_Y_1_ alloy under different deformation conditions: (**a**–**c**) 200 °C and 0.001 s^−1^; (**d**–**f**) 200 °C and 1 s^−1^.

Figure 14 shows the stress distribution of the sample under different compression conditions. Obviously, the stress distribution of the samples deformed at 200 °C and 0.001 s^−1^ is more uniform than the one deformed at 200 °C and 1 s^−1^. Nonetheless, the stress value of the samples deformed at 200 °C and 1 s^−1^ is larger than the one deformed at 200 °C and 0.001 s^−1^. This is consistent with the observation from Figure 3.

## 4. Conclusions

The constitutive equation of the Mg_98.5_Zn_0.5_Y_1_ alloy coupling with flow stress, temperature, and pulse current parameters was established. The corresponding finite element simulation using UHARD subroutine in ABAQUS software was carried out. The following results were obtained:

(1) The results obtained from the tensile tests at elevated temperatures show that the deformation temperature and strain rate are sensitive factors affecting the flow behavior of the Mg_98.5_Zn_0.5_Y_1_ alloy. Under the condition of high strain rate, the generation and propagation of dislocations are accelerated and the flow stress increases accordingly. When the deformation temperature increases, more slip systems will be activated. Hence, the flow stress inevitably decreases with the increasing temperature.
(2) The peak stress of the material decreases with the increase in current density. Compared with the current density, the effects of duty ratio and pulse frequency on the mechanical properties of the Mg_98.5_Zn_0.5_Y_1_ alloy can be almost ignored. When the current is applied, more recrystallized grains appear in the alloy and the basal texture is weakened.
(3) The constitutive equation of the Mg_98.5_Zn_0.5_Y_1_ alloy coupling with flow stress, temperature, and pulse current parameters was established. It can be obtained as follows:
  $$\dot{\varepsilon }=7.0152\times 10^{26}{\left[\sin h\left(0.0112\sigma \right)\right]}^{16.15}\exp\left(\frac{-389410}{RT}\right)\cdot \left[1-\frac{j^{2}}{j^{2}+3.15\times 10^{6}\exp\left(-8.74\times 10^{-3}T\right)}\right]$$

By comparing the experimental and simulated data, it can be concluded that the constitutive equation can predict the electrically assisted deformation of the Mg_98.5_Zn_0.5_Y_1_ alloy well. Additionally, the constructed constitutive model is implemented into ABAQUS software via a UHARD subroutine, and the results of the numerical simulation are in accord with the experimental results.

## Figures and Tables

**Table 1:** Chemical composition of the Mg_98.5_Zn_0.5_Y_1_ alloy.

Nominal Alloy	Actual Composition (at. %)			
	Mg	Zn	Y	Y/Zn
Mg_98.5_Zn_0.5_Y_1_	98.5	0.5	1	2

**Table 2:** Experimental variables and conditions of the uniaxial tensile test.

Category	Temperature T/°C	Strain Rate/s^−1^	Duty Ratio d/%	Current Density j/A∙mm^−2^	Pulse Frequency f/Hz
Thermally assisted test	200, 250 300, 350	0.001	——	——	——
	200, 250 300, 350	0.01	——	——	——
	200, 250 300, 350	0.1	——	——	——
	200, 250 300, 350	1	——	——	——
Electrically assisted test	250	0.1	22.6	44.25	30.7
	250	0.1	32.9	44.21	30.7
	250	0.1	43.5	44.35	30.7
	250	0.1	22.6	44.25	30.7
	250	0.1	22.6	44.31	58
	250	0.1	22.6	44.29	79.6
	200	0.1	22.6	26.58	30.7
	250	0.1	22.6	44.25	30.7
	300	0.1	22.6	56.74	30.7
	350	0.1	22.6	68.95	30.7

**Table 3:** Values of $j_{0}^{2}\left(T\right)$ at different temperatures.

Temperature/K	473	523	573	623
$j_{0}^{2}\left(T\right)$	145,782.6	112,759.4	63,741.62	50,132.58

## Data Availability

The data will be made available upon request.
